# The TGF-β/Smad4 Signaling Pathway in Pancreatic Carcinogenesis and Its Clinical Significance

**DOI:** 10.3390/jcm6010005

**Published:** 2017-01-05

**Authors:** Sunjida Ahmed, Azore-Dee Bradshaw, Shweta Gera, M. Zahidunnabi Dewan, Ruliang Xu

**Affiliations:** Department of Pathology, New York University School of Medicine, and Langone Medical Center, New York, NY 10016, USA; ahmeds09@nyumc.org (S.A.); azore-dee.bradshaw@nyumc.org (A.-D.B.); shweta.gera@nyumc.org (S.G.); dewanz01@nyumc.org (M.Z.D.)

**Keywords:** TGF-β, Smad4, pancreatic ductal adenocarcinoma, prognosis, therapy

## Abstract

Pancreatic ductal adenocarcinoma (PDAC) is one of the most fatal human cancers due to its complicated genomic instability. PDAC frequently presents at an advanced stage with extensive metastasis, which portends a poor prognosis. The known risk factors associated with PDAC include advanced age, smoking, long-standing chronic pancreatitis, obesity, and diabetes. Its association with genomic and somatic mutations is the most important factor for its aggressiveness. The most common gene mutations associated with PDAC include KRas2, p16, TP53, and Smad4. Among these, Smad4 mutation is relatively specific and its inactivation is found in more than 50% of invasive pancreatic adenocarcinomas. Smad4 is a member of the Smad family of signal transducers and acts as a central mediator of transforming growth factor beta (TGF-β) signaling pathways. The TGF-β signaling pathway promotes many physiological processes, including cell growth, differentiation, proliferation, fibrosis, and scar formation. It also plays a major role in the development of tumors through induction of angiogenesis and immune suppression. In this review, we will discuss the molecular mechanism of TGF-β/Smad4 signaling in the pathogenesis of pancreatic adenocarcinoma and its clinical implication, particularly potential as a prognostic factor and a therapeutic target.

## 1. Introduction

In humans, transforming growth factor β (TGF-β) plays an important role in both physiological and pathological processes. TGF-β is a cytokine that resides in the extracellular matrix and is synthesized by macrophages, lymphocytes, fibroblasts, epithelial cells, and platelets [1,2]. Physiologically, it is involved in prenatal and postnatal development, reconstruction, maintenance of normal organ structure, and wound healing [3]. TGF-β suppresses tumor formation by blocking cell cycle progression and maintaining tissue hemostasis. However, the tumor suppressive function is often lost in pancreatic adenocarcinoma by inactivation of the TGF-β signaling mediator, Smad4 [4,5,6].

Pancreatic ductal adenocarcinoma (PDAC) is one of the most aggressive cancers with a five-year survival of less than 5% due to its high recurrence rate. Surgical resection provides the patient with a chance of a cure, but only 20% of patients present early enough to be surgical candidates [7]. Moreover, in the setting of metastatic metastasis, only 5% of tumors are resectable [7]. Despite multimodality treatment with surgery and adjuvant therapy [8], the five-year survival is only increased to 20%–30% [9], thus early detection is vital in improving survival. Because the TGF-β pathway has an important role in the pancreatic carcinogenesis, many studies have focused not only on the impact of this pathway on the development of pancreatic cancers, but also on its potential clinical use in aiding in clinical management, including early detection, prognostication, and as a therapeutic option.

## 2. TGF-β Signaling

### 2.1. Smad-Dependent Pathway

**Figure 1:**
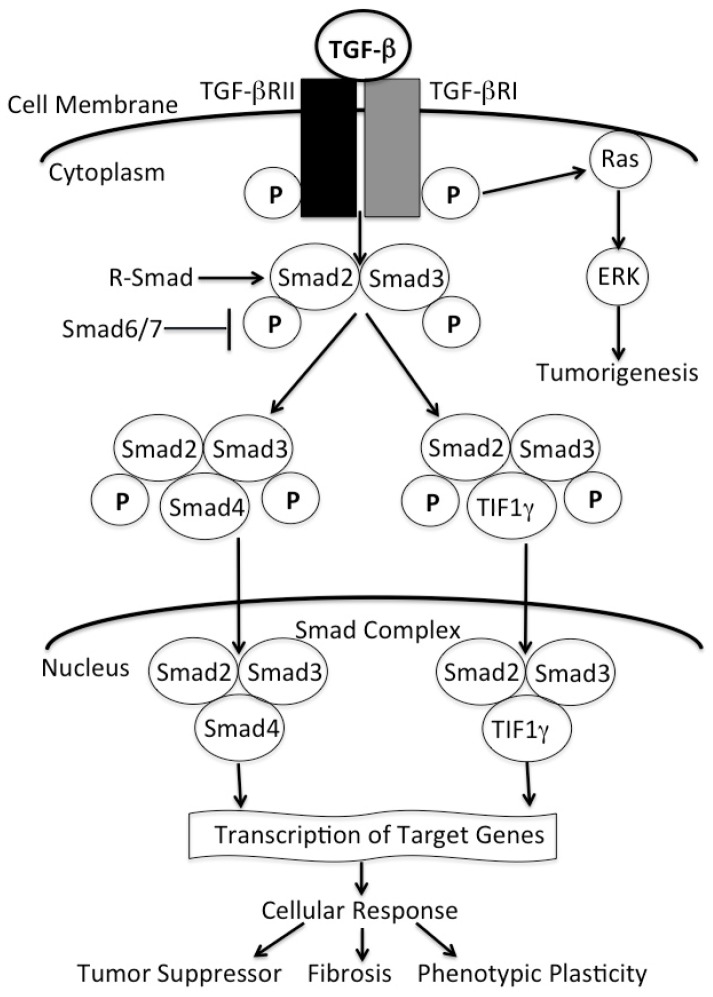
The TGF-β/Smad4 signaling pathway. The Ligand TGF-β binds a complex of transmembrane receptor serine/threonine kinases (Types I and II) in the cell surface and induces transphosphorylation of the receptors. The consequently activated receptors phosphorylate selected Smads at *C*-terminal serines, and these receptor-activated Smads (R-Smads) then form a complex with a common Smad4. Activated Smad complexes translocate into the nucleus, where they regulate transcription of target genes, through physical interaction and functional cooperation with DNA-binding transcription factors. Besides the Smad4-mediated signaling, Smad2/3 form a complex with Tiflγ and Smad complexes then translocate into nucleus, thus regulating the transcription of target genes. Activation of R-Smads by Type-I receptor kinases is inhibited by Smad6 or Smad7. Phosphorylated TGF-β receptors also activate Ras and ERK in a Smad-independent manner and induced tumorigenesis.

TGF-β, a multifunctional cytokine exists in three different isoforms in mammals (TGF-β1, TGF-β2, and TGF-β3). TGF-β1 is the most abundant and well-studied isoform. After being stimulated, active TGF-β dimers mediate signaling through TGF-β Type-I and Type-II receptors (TβRI and TβRII), which are serine/threonine kinases. The functional receptor is composed of a heterotetramer of two TβRI and two TβRII molecules [10,11]. Once this complex forms, the TβRII kinase phosphorylates a specific serine residue of TβRI, and this in turn activates the TβRI serine-threonine kinase [12]. Activation of kinase then propagates the signal transduction through phosphorylation of Smad proteins [13]. The Smad proteins include receptor-regulated Smads (R-Smad), the common mediator Smad (co-Smad), and the inhibitory Smad (I-Smad). R-Smads include Smad1, Smad2, Smad3, Smad5, and Smad8. R-Smads act as direct substrates of specific Type-I receptors, while Smad1, Smad5, and Smad8 are targets of bone morphogeneic protein (BMP) receptors. Smad2 and Smad3 are substrates of TGF-β receptors and the activin receptor, another member of the TGF-β superfamily [14,15,16]. Once phosphorylated, R-Smads associate with the common Smad, Smad4, also known as DPC4 (deleted in pancreatic cancer 4), and mediate nuclear translocation of the heterotetromeric complex (Figure 1) [17]. The Smad complex in the nucleus then regulates the expression of different genes, such as integrin, E-cadherin, collagen, and others through interaction with DNA and DNA-binding proteins [18,19]. Immediate activation of inhibitory Smads (Smad6/7) negatively regulate the signaling pathway at several levels such as TβRI degradation, Smad2/Smad3 phosphorylation, and at the level of Smad complex binding with chromatin [20]. This Smad-dependent TGF-β signaling pathway is recognized as tumor-suppressive due to the activation of cell cycle arrest, apoptosis of epithelial cells, and the maintenance of genomic integrity [21]. In pancreatic adenocarcinoma, this tumor suppressive action is often lost by the inactivation of Smad4-dependent TGF-β signaling.

### 2.2. Non-Smad-Dependent Pathway

Accumulation of TGF-β and activation of receptors activate the non-Smad pathways either by phosphorylation or by direct activation (i.e., ligand occupied receptors modulate downstream cellular response). It can be activated in parallel to Smads either downstream or upstream of Smad signaling. In Erk MAP kinase pathway, TGF-β activates Erk through activation of Ras [22,23]. Erk activation by TGF-b/ALK5 has also been shown to be mediated by the tyrosine kinase function of ALK5 [24]. Erk activation plays an important role in epithelial mesenchymal transition (EMT), which is one of the major biological functions of TGF-β [25,26]. TGF-β also induces the JNK/p38 MAPK signaling pathway. Active JNK/p38 acts in combination with the Smad pathway to regulate EMT. JNK also regulates the Smad pathway through the regulation of R-Smad, and influences TGF-β induced apoptosis [27]. Rho-like GTPases play an important role in TGF-β mediated cytoskeletal organization, cell motility, and gene expression [28]. RhoA and Rac can be activated by TGF-β via Smad-dependent or Smad-independent pathways to induce EMT. In addition, the P13/Akt pathway activated by increased TGF-β plays a role in fibroblast proliferation and morphological transformation [29].

## 3. TGF-β/Smad4 Signaling in Pancreatic Cell as a Tumor Suppressor

The TGF-β pathway is crucial in maintaining gastrointestinal homeostasis and contributes to the regulation of gastrointestinal carcinogenesis [30,31,32]. In the GI tract, TGF-β maintains homeostasis by immune modulation and suppresses tumor formation by keeping a balance between cell renewal, cell differentiation, and loss. Loss of the balance results in tumor promotion [33]. In normal pancreatic cells, TGF-β/Smad4 signaling induces a tumor suppressive effect mediated through Smad4-regulated genes [34]. Alternatively, in tumor cells from some PDAC patients, TGF-β loses its tumor suppressive effect and acts as a tumor promoter [35]. The transformation of function is due to mutations of TGF-β transduction and the loss of Smad4 signaling. TGF-β/Smad4-dependent cell cycle arrest and apoptosis in pancreatic cells is reduced as a result of the down-regulation of Smad4 in the early stage of pancreatic carcinogenesis [36]. TGF-β blocks mitogenic growth signals through Smad4, inhibiting cell growth and proliferation [37,38]. It also induces programmed cell death or apoptosis in pancreatic cell lines by regulation of TIEG, a zinc-finger gene induced by TGF-β/Smad4 signaling and acts as a tumor suppressor [39,40]. Conversely, the loss of Smad4 with a resultant increase in TGF-β aids in tumor progression through the activation of Smad4-independent signaling pathways. In advanced cancer, overexpression of TGF-β activates Ras/Erk, P13K/AKt, p38 MAPK, and Rho-GTPase pathways, which all play a role in tumorigenesis [34,41].

Over 50% of PDAC patients present with a mutation in the TGF-β pathway, with the most common mutation found in Smad4. Smad4 is located in chromosome 18q2, deletion or inactivation of which occurs in the late adenoma-to-carcinoma sequence. Homozygous deletion of Smad4 is found in about 30% of pancreatic cancer patients, inactivation of Smad4 in 20% of patients, and allelic loss of its chromosome is found in 90% [42]. Overall inactivation or loss or Smad4 is found in about 60%–90% of pancreatic adenocarcinomas [43]. Allelic loss of the Smad4 gene results in its mutation and degradation, thus triggering TGF-β independent pathways, which leads to a decrease in TGF-β cell cycle arrest and apoptosis and promotes the epithelial to mesenchymal transition [44]. However, its role in tumor progression and metastasis is complex and still under study. Studies on genetically engineered mouse models of pancreatic cancer with Smad4 mutation as well as Kras allowed a successful analysis on PDAC development and prognosis [45]. Smad4 loss alone did not initiate human pancreatic cancer formation since conditional deletion of Smad4 was not sufficient to induce either pancreatic intraepithelial lesions or invasive cancer, emphasizing the importance of multiple genetic hits [46,47,48]. Further studies have shown that Smad4 loss markedly promotes tumor development initiated by Kras G12D activation and Kras G12D/Smad4−/− tumors exhibited both increased proliferation and tumor stromal formation [46,49]. In a pancreatic cancer mouse model, Smad4−/− tumors metastasized more frequently than Smad+/+ tumors [48].

The TGF-β signaling pathway plays an important role in cancer progression. It drives progression by immune suppression, by angiogenesis, and by mesenchymal transition, which is an important factor for cancer metastasis [50,51]. EMT is a normal physiological process necessary for embryonic development and transition from epithelial cells to mesenchymal cells with expression of several mesenchymal markers such vimentin, snail, and *N*-cadherin [24,25,52,53]. In EMT, cells lose polarity and cell-to-cell contact, and acquire enhanced motility and invasiveness. TGF-β is a regulator of this transition process [54]. In pancreatic ductal adenocarcinoma, EMT is an important transition that leads to the progression and metastasis of cancer cells [50]. In the Smad4-dependent pathway, Smad3/mad4 complex induces transcription of snail protein and decreases expression of epithelial junction protein E-cadherin [55]. TGF-β also increases the expression of ZEB transcription factors through Smad4-dependent pathways, thus further increasing the EMT response [56]. In the late stage of tumorigenesis, TGF-β promotes tumor growth by a combined effect of Smad4-dependent and -independent effects on EMT [57,58]. These abovementioned activities emphasize that TGF-β induces pancreatic cancer progression not only by immunosuppression but also, and more importantly, by EMT [51].

Another way of promoting metastasis in cancer is through angiogenesis, and TGF-β acts on endothelial cell proliferation and migration, as well as capillary formation and thereby angiogenesis, promoting vascular metastasis [59]. A major factor for this vascularization is vascular endothelial growth factor (VEGF), which is induced by TGF-β. The major stimulation for this expression is hypoxia, which is a common microenvironment in a growing tumor [59]. TGF-β promotes the secretion of proangiogenic factors, such as matrix-metalloproteinases2 (MMP2) and MMP-9, and downregulates the expression of anti-angiogenic factors such as protease inhibitor TIMP [10,21,60,61] through the recruitment of inflammatory cells in the tumor environment [59]. TGF-β further promotes angiogenesis by inducing connective tissue growth factor (CTGF) in addition to VEGF [10,60,62].

In addition to its effect on tumor epithelial cells, TGF-β modulates tumor development and progression by changing the tumor microenvironment [63]. TGF-β suppresses immune and inflammatory processes through the inhibition of CD+ cytotoxic T cells, macrophages, dendritic cells, and NK cells [63]. These changes lead to an elevated TGF-β in 50% of TGF-β mutated PDAC [64]. In PDAC, calcium binding inflammatory proteins, S100A8/A9, are overexpressed by tumor-infiltrating inflammatory cells as well as in PDAC cells [65,66,67]. Smad4 depletion is associated with reduced S100A8 positive infiltrating inflammatory cells [65,66]; conversely, these proteins are expressed by PDAC cell lines only in areas of Smad4 inactivation [67]. It has recently been found that inflammatory transcription factor NF-κB activation in PDAC is closely involved in driving tumor progression, especially when its activation is sustained. Thus, Smad4 plays a key role in linking inflammation and cancer [68]. Intact Smad4, S100A8, S100A9, and S100A8/A9 share an overall inhibitory effect on NF-κB, while these molecules do not affect NF-κB in the presence of Smad4 homozygous deletion.

Deposition of extracellular matrix (ECM) or desmaplastic reaction is a hallmark of PDAC. ECM is composed of structural proteins such as fibronectin collagen [69,70]. In addition to ECM, endothelial cells, immune cells, and the fibroblast of tumor microenvironments contribute to tumor growth invasion and chemoresponse [71,72]. TGF-β expression enhances the release of multiple ECM molecules including fibronectin, collagen fibulins, and elastin [73,74]. Overexpression of TGF-β is a major factor of fibrosis in many tumors [2,75,76]. In cell culture conditions, TGF-β stimulates proliferation of fibroblast in skin and lung cells and increases collagen synthesis in pancreatic and liver cells [1,70,74]. TGF-β also inhibits degradation of newly synthesized ECM by inhibiting the synthesis of MMP and by inhibiting the expression of genes responsible for the production of MMP [77].

## 4. Transcriptional Intermediary Factor 1 Gamma (Tif1γ) in the Regulation of the TGF-β Pathway

Tif1γ (or Ectodermin/PTC/RFG7/TRIM33) is a transcriptional cofactor competing with Smad2/Smad3 for binding to Smad4, or targeting Smad4 for degradation, although its role in carcinogenesis is unclear. It has a critical role in regulation of the TGF-β signaling pathway and can have a positive or negative role in this pathway as shown in the literature. It can act as a negative regulator of the pathway by controlling Smad4 function. Smad4 regulation is the biological target of Tif1γ. It is shown in the mouse pancreatic model that tumor suppressive effects of Tif1γ could be independent of Smad4 [78]. Overexpression of Tif1γ can lead to inhibition of the TGF-β signaling pathway [79,80]. Tif1γ antagonizes transcriptional response of TGF-β by forming a complex with Smad4, also shown in pancreatic cell line [79,81]. Tif1γ affects the stability of Smad4 by causing its degradation by ubiquitin-proteasome pathway in human and Xenopus cells [79]. Tif1γ also regulates localization of Smad4 (nuclear versus cytoplasmic) and its depletion leads to nuclear localization of Smad4 [79]. Ligr et al. showed inverse relationship between levels of Tif1γ and Smad4 in the pancreatic cells [82].

A study has demonstrated that Tif1γ could cause up-regulation of the TGF-β signaling pathway by acting through Smad4-independent pathway and competing with Smad4 to bind with phosphorylated Smad2/3 to transduce signals [83]. Tif1γ has a significant role in pancreatic carcinogenesis. Recent studies have shown its role as a tumor suppressor in pancreatic cancer. Tif1γ expression has shown to be decreased in pancreatic ductal adenocarcinomas with the use of RT-PCR and immunohistochemistry [84] Tif1γ inactivation in the presence of activated Kras mutation can result in cystic pancreatic tumors in the mouse model [84]. Ligr et al showed imbalanced expression of Tif1γ has anti-proliferative effect on pancreatic ductal epithelial cells. Tif1γ overexpression as well as under expression can result in inhibition of pancreatic cell growth by arresting cell cycle.

## 5. TGF-β/Smad4 as a Prognostic Marker of PDAC

TGF-β level is elevated in the tissue and plasma of PDAC. Plasma levels of TGF-β are correlated with the presence of metastases in CRC, PDAC, and some non-gastrointestinal tumors such as prostate and breast cancers [85]. The association of Smad4 inactivation with poor prognosis may relate to an increased propensity of PDAC to metastasize widely [86]. A recent study conducted on 76 rapidly autopsied patients with PDCA found that Smad4 negative patients had widespread metastasis [87]. Similarly, colorectal cancer with Smad loss was associated with progression to metastasis [88].

A study was done to assess the prognostic significance of the TGF-β level in PDAC patients. TGF-β level was determined in the serum of 146 PDAC patients and 58 patients with benign pancreatic conditions. In healthy controls, serum levels of TGF-β were 57.6 ± 23.2 ng/mL (mean ± SD); in benign pancreatic conditions, it was 64.5 ± 27.4 ng/mL; in PDAC, it was 237 ± 45.3 ng/mL. In this study, they found that serum levels of TGF-β were significantly higher in PDAC patients than patients with benign pancreatic conditions. They also concluded that high levels of TGF-β were associated with increased tumor size, metastasis (lymphatic and distant), and higher tumor stage [89]. The five-year median overall survival was 21.7% in the low TGF-β group and 15.5% in the high TGF-β group (*p* < 0.01) [89]. The data demonstrate that an elevated level of TGF-β decreases the patient survival rate. Investigators also proved that patients with elevated TGF-β correlated with the risk of death. The correlation between TGF-β and increased invasion of pancreatic cancer was also observed in several studies. Accumulated data support that PDAC patients with high TGF-β levels have an increased risk of metastasis and poor prognosis, and the level of TGF-β can be used as a prognostic marker [89].

Many studies have shown that loss or inactivation of SMAD4 is associated with poor prognosis. In a study of more than 200 patients with PDAC, intact expression of SMAD4 detected by immunohistochemistry was associated with a significantly improved median survival and five-year survival (19.2 months and 20.5 months for intact expression compared to 14.7 months and 13.7 months for loss of expression, respectively). In the multivariate Cox model, SMAD4 status was an independent prognostic factor [90]. A meta-analysis of 4247 patients in 20 published articles concluded that the immunohistochemical loss of SMAD4 predicted a poor overall survival in both Asian and Caucasian patients with pancreatic cancer, but did not correlate with tumor size, differentiation, or lymph node metastasis [91]. Another meta-analysis of 1762 patients from 14 studies found that loss of SMAD4 correlated significantly with poor overall survival. The multivariate analysis showed that the loss of SMAD4 predicted poor prognosis in patients with less advanced disease (likely Stage I to Stage II pancreatic cancer) [92].

## 6. TGF-β/Smad4 as a Therapeutic target for PDAC

All of these abovementioned studies have supported a strong association of TGF-β with the development of pancreatic adenocarcinoma (50% of PDAC is due to mutations of TGF-β), metastasis, and prognosis. This would make TGF-β a potential therapeutic target for PDAC [69]. PDAC mouse models have shown that TβRII neutralization could reduce the metastasis and proliferation of cancer cells significantly while increasing apoptosis in the primary tumor [69].

TGF-β signaling through TβRII is a prerequisite pathway for tumor cells. The neutralization of TβRII with a monoclonal antibody 2G8 resulted in a decrease in fibroblast maturation and collagen deposition. It also changes the tumor microenvironment by increasing the epithelial differentiation more than mesenchymal differentiation, thereby reducing metastasis [69]. This strongly suggests that the 2G8 monoclonal antibody has a therapeutic potential for PDAC.

Several studies have demonstrated that TGF-β can mediate responses through a Smad-independent pathway, and that some of these responses are found in conjunction with increased expression of TβR and TGF-β isoforms in pancreatic cancer [93,94]. In a different study on Smad4 deficient PDAC cell lines, PDAC cells displayed constitutive activation of the TβR system as a result of autocrine production and activation of TGF-β [4]. The study demonstrates that PDAC cell lines have escaped the tumor suppressive function and constitutively elevated the level of phosphorylated R-Smads (pSmad4), which is dependent on the rate of TβRI kinase [95,96]. In an in vitro study, the investigators observed that constitutive activation of endogenous TGF-β receptor signaling drives cell migration and invasion in a cell-autonomous manner. When the cell lines were treated with TβRI kinase inhibitor, SD-093, they found significant inhibition of cellular migration and invasiveness, whereas treatment of the same cell lines with exogenous TGF-β further stimulates their invasiveness in vitro [4]. All of these findings highlight a potential of targeting TβRI kinase to treat an aggressive subtype of PDAC.

The therapeutic significance of Smads is unclear. Although inactivation or loss of Smad4 occurs in the majority of pancreatic cancer, targeting Smad4 or other Smads as treatment of PDAC may not be successful due to presence of the Smad-independent TGF-β signaling pathway.

## 7. Conclusions

Current research has shed light on the biological pathways of TGF-β and its role in carcinogenesis. The TGF-β signaling pathway is involved in tumor suppression and promotion, through the activation of early and late genes, but the complicated mechanism of transition from precancerous cells to cancerous cells is still unknown, particularly in pancreatic carcinogenesis. It is known that TGF-β is a growth factor for gastrointestinal tract and pancreatic cancer, similar to EGFR in non-small cell lung cancer. Elevated levels of TGF-β are found in noncancerous pancreatic tissue and in tumor tissue, and studies have shown that it may serve well as a marker for tumor progression and poor survival in patients with PDAC [97]. There are a myriad of proteins including Smads and Tiflγ that TGF-β interacts with in the signal transduction pathways that promote or inhibit cancer growth. The findings imply that TGF-β and its signaling pathway may be a potential target of therapy and information related to this signaling pathway can be helpful in management of patients with PDAC.
